# The functional connectivity of the basal ganglia subregions changed in mid-aged and young males with chronic prostatitis/chronic pelvic pain syndrome

**DOI:** 10.3389/fnhum.2022.1013425

**Published:** 2022-09-30

**Authors:** Xi Lan, Xuan Niu, Wei-Xian Bai, Hai-Ning Li, Xin-Yi Zhu, Wen-Jun Ma, Jian-Long Li, Wang-Huan Dun, Ming Zhang, Juan He

**Affiliations:** ^1^Department of Rehabilitation Medicine, The First Affiliated Hospital of Xi’an Jiaotong University, Xi’an, China; ^2^Department of Medical Imaging, The First Affiliated Hospital of Xi’an Jiaotong University, Xi’an, China; ^3^Department of Medical Imaging, Xi’an No.3 Hospital, Xi’an, China; ^4^Department of Urology, Xi’an No.3 Hospital, Xi’an, China

**Keywords:** basal ganglia, resting-state functional MRI, functional connectivity, machine learning, chronic prostatitis/chronic pelvic pain syndrome (CP/CPPS)

## Abstract

**Background:**

The Basal ganglia (BG) played a crucial role in the brain-level mechanisms of chronic pain disorders. However, the functional changes of BG in chronic prostatitis/chronic pelvic pain syndrome (CP/CPPS) are still poorly understood. This study investigated the BG subregions’ resting-state functional connectivity (rs-FC) in CP/CPPS patients compared with healthy controls.

**Methods:**

Twenty eight patients with CP/CPPS and 28 age- and education-matched healthy males underwent clinical measurements and 3T brain MR imaging, including T1-weighted structural images and resting-state functional imaging. The data were analyzed by the seeded-based rs-FC analysis. Then, a machine learning method was applied to assess the feasibility of detecting CP/CPPS patients through the changed rs-FC.

**Results:**

Compared with healthy males, patients presented decreased rs-FC between the BG subregions and right middle cingulate cortex, and correlated with pain (*r* = 0.51, p-uncorrected = 0.005) and urinary symptoms (*r* = –0.4, p-uncorrected = 0.034). The left superior temporal gyrus and right supramarginal gyrus showed decreased rs-FC with the BG subregions as well. The area under the receiver operating characteristic curve of 0.943 (accuracy = 80%, F1-score = 80.6%) was achieved for the classification of CP/CPPS patients and healthy males with support vector machine (SVM) based on the changed rs-FC.

**Conclusion:**

These findings provide evidence of altered BG subregions’ rs-FC in CP/CPPS, which may contribute to our understanding of the BG’s role in CP/CPPS.

## Introduction

Chronic prostatitis/chronic pelvic pain syndrome (CP/CPPS) is one of the most common urologic diseases, with the prevalence rate ranging from 3 to 16% all over the world (Engeler et al., 2013). CP/CPPS affects 8.4% of men in China, especially those between 31 and 40 years old (Liang et al., 2009). The persistent pain and urinary symptoms severely bother the patients and urologists. The key to breaking this situation is a better understanding of the ambiguous pathophysiological mechanisms (Pontari, 2013). Nowadays, neuroimaging studies have led to advances in comprehending CP/CPPS mechanisms at the brain level beyond the prostate. Previous studies assessed alterations in brain structure or function in the patients with CP/CPPS, such as medial areas of the motor-sensory cortex, the posterior insula, and the periaqueductal gray, which indicated the abnormal sensorimotor processing of the pelvic area and descending modulation of pain in this chronic disorder (Clemens et al., 2019).

In chronic pain conditions, the basal ganglia (BG) were supposed to integrate many aspects of pain. These include the integration of motor, emotional, autonomic, and cognitive responses to pain (Borsook et al., 2010). The multiple painful stimuli produced increased or decreased activation in the subregions of BG (Becerra et al., 2006, 2015; Lebel et al., 2008), which implicated that BG were involved in nociceptive signal processing. The increase of BG gray matter has been reported in chronic back pain (Tagliaferri et al., 2021), fibromyalgia (Schmidt-Wilcke et al., 2007), and chronic vulval pain (Schweinhardt et al., 2008), suggesting these structural changes might be associated with some underlying functional alterations in chronic pain conditions. In complex region pain syndrome (CRPS) (Becerra et al., 2014; Azqueta-Gavaldon et al., 2020), the decreased functional connectivity (FC) of BG with sensorimotor network and Default Mode Network (DMN) were involved a maintained fear of pain and movement avoidance, and the information integration and environment perception in pain processing. In contrast, females with chronic pelvic pain presented increased FC between BG and posterior cingulate cortex, another hub of the DMN (Martucci et al., 2015). In clinical practice, BG were a target region for chronic pain treatment. The deep brain stimulation at the globus pallidus could improve the chronic pain symptoms in Parkinson’s disease (Gong et al., 2020). These results highlighted the important role of BG in pain processing. In particular, A whole-brain analysis between CP/CPPS and healthy controls (HCs) compared FC effect size directly and found the difference in the BG subregion greater than 1 in magnitude (Kutch et al., 2015). However, it remains unclear concerning the functional properties of BG and subregions in males with CP/CPPS.

Herein, in this MRI-based research, we hypothesized that the resting-state functional connectivity (rs-FC) of the BG subregions was altered in patients with CP/CPPS and investigated the relationship between the altered rs-FC and clinical measures of CP/CPPS. And, we explored the feasibility of detecting CP/CPPS by the support vector machine (SVM) algorithm based on the altered rs-FC.

## Materials and methods

This study was performed from April 2020 to August 2021. The patients with CP/CPPS were recruited from the urology outpatient department, and age- and education- matched healthy males were recruited as healthy controls in this study. The written informed consents were obtained from all participants and conducted following the Declaration of Helsinki.

### Participants

Twenty-eight patients with CP/CPPS (31.18 ± 4.33 years, mean ± *SD*) and 28 demographically similar HCs (30.11 ± 2.91 years, mean ± *SD*) were included in this study. The inclusion and exclusion criteria of all subjects was shown in Table 1. The diagnosis of CP/CPPS was established after history-taking, physical examination, laboratory examination, and kidney, ureter, bladder, and prostate ultrasound, and consistent with the National Institutes of Health definition of CP/CPPS. The clinical profiles of patients with CP/CPPS were shown in Supplementary Table 1.

**TABLE 1:** Inclusion and exclusion criteria.

	Inclusion criteria	Exclusion criteria
CP/CPPS	(a) aged from 18 to 40 years; (b) diagnosed with CP/CPPS; (c) symptoms present for a majority of the time during the most recent 3 months, or most of the time during the most recent 3 months; (d) NIH-CPSI total score ≥ 12 and pain score > 0; (e) right-handed.	(a) contraindications to performing MRI; (b) had a history of surgery, deformity, infection, or cancer in the genito-urinary system; (c) a history of psychiatric disorders, substance abuse, treatment with antidepressants or other medications; (d) other organic diseases or chronic pain in other body sites.
HCs	(a) aged from 18 to 40 years males; (b) no chronic pain in pelvic or bladder region, and chronic pain in other body region; (c) right-handed.	

### Questionnaires

The National Institutes of Health Chronic Prostatitis Symptom Index (NIH-CPSI) was applied to assess the severity of CP/CPPS symptoms (Litwin et al., 1999). The NIH-CPSI consists of nine items divided into three domains, (1) the severity of pain or discomfort include the location, intensity, and frequency (4 items, 0–21 points); (2) the severity of urinary symptoms focus on irritative and obstructive urinary symptoms (2 items, 0–10 points); and (3) the quality-of-life (QoL) evaluate the symptoms effect on ordinary life (3 items, 0–12 points). The higher three sub-scores (pain, urinary symptoms, and QoL) and the total score (0–43 points) indicate worse symptoms.

All the subjects were assessed for catastrophic thoughts through the Pain catastrophizing Scale (PCS) (Xu et al., 2015). The PCS is a 13-item self-report measure designed to assess catastrophic thoughts or feelings accompanying the experience of pain. Which can be divided into the three components of pain catastrophizing: rumination (e.g., “I can’t seem to keep it out of my mind”); magnification (e.g., “I wonder whether something serious may happen”); and helplessness (e.g., “There is nothing I can do to reduce the intensity of pain”). This scale has been translated and standardized in Chinese population. Participants rated in reference to a previous pain event on a 5-point Likert scale ranging from 0 (not at all) to 4 (always).

### Neuroimaging data acquisition

All participants underwent MR scans on a 3T MR scanner (Ingenia, Philips Healthcare, Best, Netherlands) equipped with a 32-channel head coil. All participants were asked to empty their bladder and lie still in a relaxed position with their eyes closed while remaining awake and avoiding specific thoughts. Headphones and foam pads were used to reduce noise interference and minimize head motion. Routine sequence scanning was first performed to exclude obvious structural damage. High-resolution brain structural images were acquired from each subject [repetition time (TR) = 8.2 ms, echo time (TE) = 3.8 ms, flip angle = 8°, slice thickness = 1 mm, field of view (FOV) = 24 × 24 cm^2^, matrix size = 240 × 240, voxel size = 1 × 1 × 1 mm^3^]. The rs-fMRI images were obtained from gradient-echo-planar imaging sequence (TR = 2,500 ms, TE = 30 ms, flip angle = 90°, voxel size = 3 × 3 × 3 mm^3^, slice thickness = 3 mm with no gap, slices = 50, matrix size = 80 × 80, FOV = 24 × 24 cm^2^, volumes = 180).

### Image preprocessing

All neuroimaging data were processed by using CONN connectivity toolbox V20.a^1^ (Whitfield-Gabrieli and Nieto-Castanon, 2012), based on MATLAB 2019. The structural and functional data underwent the preprocessing pipeline for volume-based analyses, including (1) removing the first four volumes to avoid the potential noise related to the participants’ adaptation to the scanner, (2) functional realignment and unwarping, the subjects with head motion exceeding 3 mm or 3^°^ were excluded, and no participants met this exclusion criterion. (3) functional slice-timing correction, (4) functional outlier identification (ART-based scrubbing), (5) functional segmentation and normalization to Montreal Neurological Institute (MNI) space, (6) structural segmentation and normalization in the MNI -space, (7) functional smoothing by using a Gaussian kernel with full-width at half-maximum of 6 mm. For the scrubbing, outliers were defined as composite movement greater than 0.5 mm or more than 3 standard deviations away from the mean image intensity. Denoising was processed by the usual covariates (white-matter and cerebrospinal fluid signals) and band-pass filtering with the default CONN values (0.008–0.09 Hz). The comparison of the imaging quality and head motion showed no significant difference between two groups (Supplementary Figure 1). Finally, head motion-related artifacts were reduced by the component-based noise correction method (CompCor).

### Seed-based functional connectivity analysis

A seed-based analysis was performed to explore the whole-brain voxel-wise rs-FC alteration in the BG subregions between two groups. The bilateral BG subregions were defined based on the Harvard-Oxford Brain Atlas per CONN Toolbox protocol (Whitfield-Gabrieli and Nieto-Castanon, 2012). A total of 8 BG subregions were defined as the region of interest (ROIs) for the following analysis, including the bilateral caudate nucleus (NC), globus pallidus (GP), nucleus accumbens (NAc), and putamen (PU). The time course of the average blood oxygen level-dependent (BOLD) signal was extracted from each ROI. Then, Pearson correlation coefficients were calculated between the mean time series of each ROI and that of each voxel of the whole brain. Fisher z transformation was performed to improve the normal distribution of the data. The two-sample *t*-tests were used to group comparisons of seed-based rs-FC. The results were considered significant at a threshold of voxel-wise *p* < 0.001 and cluster-level *p* < 0.05, with the false positive rate corrected (FDR-corrected) for between-group comparisons. The resulting maps were overlaid on the rendered views using MRIcron,^2^ and the location of the surviving brain regions was reported by the automated anatomical labeling (AAL) template (Tzourio-Mazoyer et al., 2002).

### Statistical analysis

Two-sample *t*-tests or Mann–Whitney *U*-tests were used to evaluate the differences in age, and PCS scores according to the normality and homogeneity of variances assessed by Kolmogorov–Smirnov and Levene’s tests, respectively. The significant level of non-imaging data was set to *p* = 0.05. Statistical analyses were performed with R (R version 4.0.2, The R Foundation for Statistical Computing).

### Correlation analysis

Compared to HCs, the rs-FC with significant CP/CPPS group changes was extracted to explore the relationship with clinical features and the score of questionnaires, respectively. Then, the Spearman correlation analysis was performed by using GraphPad Prism 9 to evaluate the correlation between abnormal rs-FC and changed measurements in patients with CP/CPPS. The FDR method was used to correct the results of the correlation analysis for multiple comparisons.

### Machine learning

Based on the altered rs-FC, classification models of CP/CPPS were established by using the SVM. We adopted nested resampling and stratification sampling strategies to reduce selection bias and avoid overfitting or underfitting (Supplementary Figure 2). Fivefold cross-validation (CV) were adopted to get different training and testing data sets (outer resampling). As for the training data, we used the 10-fold CV to get different inner training and testing data sets (inner resampling). Then, a grid search was performed to determine the optimal parameter for the model tuning within the inner resampling. The learners with the tuned hyperparameter configuration obtained in the inner resampling were applied to the outer training data set. The classification performance of the machine learning algorithms was evaluated by the accuracy, F1-Score, and area under the receiver operating characteristic curve (AUC-ROC). The classification measurement of outer training data was calculated and taken as the average to evaluate the generalization ability of the classification model.

We have trained the machine learning model for significant rs-FC of each seed independently. And then, to see whether we can get better performance with all these seeds, we trained the model again by combing the rs-FC of all seeds. The machine learning was performed in R version 4.0.2 and the Machine Learning in R package (MLR3) (Lang et al., 2019).

## Results

### Demographic and clinical data

Compared to HCs, no significant differences were found for age (*p* = 0.201), and education (*p* = 0.386). The PCS total scores (*p* = 0.018) and three sub-scores (Rumination *p* = 0.007, Magnification *p* = 0.031, Helplessness *p* = 0.093) were higher in CP/CPPS group. In the CP/CPPS group, the total score, pain sub-score, urinary symptoms sub-score, and QoL sub-score of NIH-CPSI were 21.29 ± 4.78, 10.25 ± 2.52, 3.07 ± 3.06, and 7.96 ± 2.82 (mean ± *SD*), respectively. The disease duration was 21.75 ± 18.12 months (mean ± *SD*). The detailed information is listed in Table 2.

**TABLE 2:** Demographical, clinical data of CP/CPPS and healthy controls.

	CP/CPPS (*n* = 28)	HCs (*n* = 28)	*P*-value
**Age (years)**	31.18 ± 4.33	30.11 ± 2.91	0.201
**Education (years)**	15.39 ± 1.68	15.35 ± 1.80	0.386
**Duration (months)**	21.75 ± 18.12	–	–
**NIH-CPSI**			
Total	21.29 ± 4.78	–	–
Pain subscore	10.25 ± 2.52	–	–
Urinary subscore	3.07 ± 3.06	–	–
QoL	7.96 ± 2.82	–	–
**PCS**			
Total	19.79 ± 7.57	13.29 ± 10.41	0.018
Rumination	8.50 ± 3.11	5.71 ± 3.74	0.007
Magnification	4.96 ± 1.86	3.43 ± 2.82	0.031
Helplessness	6.32 ± 4.51	4.32 ± 4.13	0.093

Unless otherwise indicated, data are mean ± *SD*. CP/CPPS, chronic prostatitis/chronic pelvic pain syndrome; HCs, healthy controls; NIH-CPSI, The National Institutes of Health chronic prostatitis symptom index; QoL, the quality of life; PCS, the Pain catastrophizing Scale.

### Seed-based functional connectivity analysis

**FIGURE 1:**
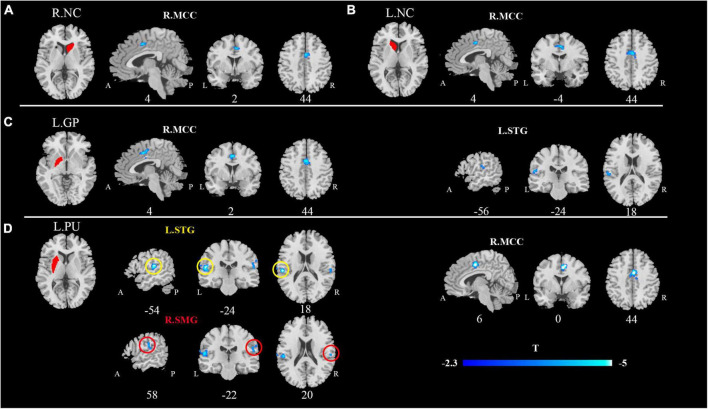
Decreased functional connectivity of CP/CPPS in contrast to healthy controls between the seed regions. **(A)** R.NC; **(B)** L.NC; **(C)** L.GP; **(D)** L.PU and affected brain regions. All images were shown with an FDR correction of *P* < 0.05. R, right; L, left; NC, Caudate nucleus; PU, putamen; GP, Globus Pallidus.

Compared with HCs, patients with CP/CPPS exhibited significantly decreased rs-FC between the bilateral NC and the right middle cingulate cortex (R.MCC) (Figures 1A,B). Meanwhile, the left GP (L.GP)-R.MCC/left superior temporal gyrus (L.STG) was presented with decreased rs-FC in CP/CPPS group (Figure 1C). The rs-FC of L.PU- L.STG/R.MCC/right supramarginal gyrus (R.SMG) was reduced in the patients with CP/CPPS (Figure 1D and Table 3). There was no significant change in rs-FC of other seeds (R.PU, R.GP, and bilateral NAc).

**TABLE 3:** The seed-based functional connectivity analysis.

Comparisons	Seeds	Area	Cluster size		Peak MNI coordinate		
					
			Voxels	*t*	*x*	*y*	*z*
HC > CP/CPPS	R.NC	R. MCC	177	4.49	4	2	44
	L.NC	R. MCC	240	4.56	4	–4	44
	L.PU	L. STG	340	5.98	–54	–24	18
		R. MCC	492	5.72	6	0	44
		R. SMG	302	4.61	58	–22	20
	L.GP	R. MCC	397	4.73	4	2	44
		L. STG	122	4.4	–56	–24	18

MNI, Montreal Neurological Institute; R, right; L, left; NC, Caudate nucleus; PU, putamen; GP, Globus Pallidus; MCC, Middle Cingulate Cortex; STG, Superior Temporal Gyrus; SMG, Supramarginal gyrus.

### Correlation analysis

**FIGURE 2:**
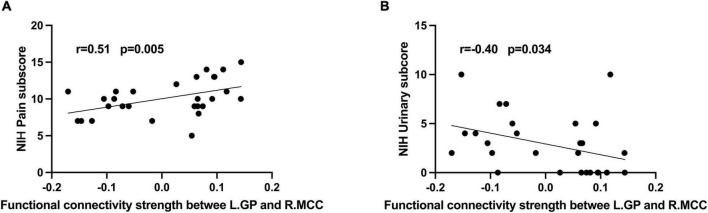
Correlation analysis between **(A)** rs-FC of L.GP- R.MCC and NIH-CPSI pain score (*r* = 0.51, *P* = 0.005); **(B)** rs-FC of L.GP- R.MCC and NIH-CPSI urinary score (*r* = –0.40, *P* = 0.034).

In CP/CPPS group, L.GP-R.MCC rs-FC were positively correlated with pain sub-score (Figure 2A, R = 0.51, *p* = 0.005) and negatively correlated with urinary symptoms sub-score (Figure 2B, R = –0.4, *p* = 0.034). However, these correlations were statistically insignificant after multiple FDR comparisons. In addition, there were no significant correlations between altered rs-FC and other measurement values in the CP/CPPS group.

### Classification performance by machine learning

**FIGURE 3:**
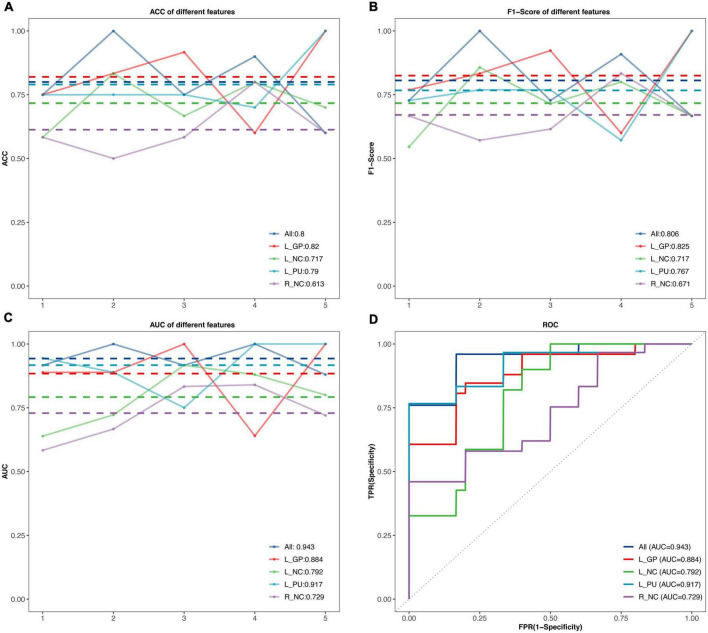
The classification performance of the SVM model. **(A–C)** The accuracy, F1-Score, and the area under curves (AUC) values in the testing set for different features. The horizontal axis represents five outer resampling, the vertical axis represents the values of measurement. The mean value of each measurement is marked with the dotted line of the same color as the broken line. **(D)** The receiver operating characteristic (ROC) curves. ACC, accuracy; AUC, area under the curve; R, right; L, left; NC, Caudate nucleus; PU, putamen; GP, Globus Pallidus.

The performance of SVM classifiers based on different features (rs-FC of L.GP, L.NC, L.PU, R.NC, and all seeds) at each outer resampling was shown in Figures 3A–C. CP/CPPS patients were classified with mean accuracy of 82% (L.GP), 71.7% (L.NC), 79% (L.PU), 61.3% (R.NC), and 80% (all seeds). The mean F1-Score of the classifiers were 82.5% (L.GP), 71.7% (L.NC), 76.7% (L.PU), 67.1% (R.NC), and 80.6% (all seeds). The area under the AUC-ROC was 0.884 (L.GP), 0.792 (L.NC), 0.917 (L.PU), 0.729 (R.NC), and 0.943 (all seeds) (Figure 3D). The SVM based on the combination of all the seeds achieved a satisfying performance, especially the largest AUC-ROC.

## Discussion

This study investigated the rs-FC of BG subregions and their relationship with clinical features in mid-aged and young CP/CPPS patients. Compared to the HCs, CP/CPPS patients presented decreased connectivity of BG subregions (bilateral NC, L.PU, L.GP) and MCC. Meanwhile, L.PU and L.GP showed reduced connectivity with the left STG. We also found lowered rs-FC of L.PU with R.SMG in CP/CPPS patients. Furthermore, correlations analysis indicated the decreased rs-FC of L.GP-R.MCC was positive with pain and negative with urinary symptoms. Based on these alterations, we established SVM classifiers for distinguishing CP/CPPS patients from HCs. The SVM based on the all altered rs-FC of BG subregions to identify CP/CPPS achieved the largest ROC-AUC.

As we know, this is the first study to examine the rs-FC of BG subregions in young and mid-age males with CP/CPPS. We found the reduced rs-FC of NC, L.PU, and L.GP with MCC in CP/CPPS. As a central hub in pain processing, MCC was connected with other brain areas involved in the processing of pain (Vogt, 2005; Eisenberger, 2012). In chronic pain conditions, such as refractory neuropathic pain (Maarrawi et al., 2007), fibromyalgia syndrome (Pérez-Aranda et al., 2019), and chronic low back pain (Ivo et al., 2013), MCC presented structural and functional abnormalities. The MCC was associated with pain intensity and motor responses toward nociceptive (Shackman et al., 2011). A graph theory study found that patients with CP/CPPS presented disrupted topological organizations of MCC, the higher the global and local efficiency of MCC, the more serious chronic pain (Huang et al., 2021a). In our study, the correlation analysis showed decreased rs-FC of L.GP-MCC was a positive correlation with pain symptoms in CP/CPPS patients. In addition to being involved in pain processing, MCC generated the desire to void and urinary urgency (Griffiths, 2015). We also found the urinary symptoms of CP/CPPS were negatively correlated with rs-FC of L.GP-MCC. These results may implicate that the detaching between BG and MCC affects the clinical symptoms of CP/CPPS.

The BG-cingulate circuit is one of the BG-thalamocortical circuits (Alexander et al., 1986). A Diffusion Tensor Imaging (DTI) study revealed that patients with CP/CPPS presented microstructural differences within the BG-thalamocortical circuits (Woodworth et al., 2015). The striatum (NC and PU) is the input nucleus and receives signal inputs from the multiple cortexes, the GP is the output nucleus and from there back to the associated cortical area (Marchand, 2010). The sensory and motor control progressively integrated with their subsequent passage through BG (Borsook et al., 2010). Thus, the BG is considered to be involved in the modulatory system of pain (Barceló et al., 2012; Becerra et al., 2015). Similar to our finding, the temporomandibular disorder patients showed reduced rs-FC of BG-cingulate, which was positively correlated with the pain intensity (He et al., 2018). BG is densely populated with opiate receptors in humans (Blackburn et al., 1988). Especially in the BG-cingulate circuit, the inhibition of the cingulate cortex or BG could relieve pain in the rodent model of chronic pain (Zhuang et al., 2021). In the present study, we found decreased L.GP-MCC rs-FC was a positive correlation with pain symptoms in CP/CPPS patients. The reduced rs-FC of BG-cingulate might be a compensatory coping strategy in chronic pain conditions. The compensation strategy seems to lose efficacy for individuals with more serious pain. So, in this study, the reduced rs-FC within the BG-cingulate circuit may be associated with aberrant central control in pain processing in patients in CP/CPPS.

We also found that L.STG and R.SMG presented decreased rs-FC with L.PU and L.GP. The R.SMG belongs to the ventral attention network (VAN), which supports bottom-up attention to important, behaviorally relevant stimuli, even if they are not salient or distinctive (Power et al., 2011). The STG is generally considered to be important as auditory perception and emotional regulatory part of the human brain, which is essential for individual stressful experiences, cognitive processes, and adaptive behavior (Huang et al., 2021b). A few neuroimaging studies about pain found that the function or structure of SMG (Yu et al., 2019; Ionta et al., 2020; Naor et al., 2020; Yang et al., 2021) and STG (Cleve et al., 2017; Zhao et al., 2017; van Ettinger-Veenstra et al., 2019; Zhang Y. et al., 2019; Zhang Y. N. et al., 2019; Spisak et al., 2020; Yang et al., 2020) was changed. The pain experience results from the integration of pain processing in a given individual (Wiech et al., 2008), our finding might partly reflect this integration. However, how these brain regions affect pain is incompletely understood, it needs to be further investigated.

Machine learning has been widely used to identify various chronic pain as an auxiliary method for diagnosis and prediction in neuroimaging studies. Based on the amplitude of low-frequency fluctuation within the region of interest (ROI), the researchers trained machine learning models that could discriminate chronic low back pain patients from HCs and reach the highest accuracy of 71.1% (Zhang Y. et al., 2019). The rs-FC was selected as an input feature of SVM that could differentiate irritable bowel syndrome patients with a ROC-AUC of 0.71 (Mao et al., 2020). An SVM classifier based on the changed brain morphology to identify patients with chronic pelvic pain achieved an accuracy of 73% (Bagarinao et al., 2014). In the present study, we combined all altered rs-FC of BG subregions to build the SVM classifier, which achieved the highest ROC-AUC and satisfactory performance. The altered rs-FC of BG subregions had an advanced ability to objectively classify individuals with CP/CPPS and played an important role in CP/CPPS mechanisms at the brain level.

Our study had several limitations. First, our research has a cross-sectional design with a relatively modest size. Second, this study did not examine other precise structures of BG, such as nucleus subthalamic and substantia nigra pars. Finally, the nested resampling approach was used to construct and tune the SVM model due to the small sample size, which could cause the overfitting issue. Further studies with larger sample sizes should be extended to these subdivisions to investigate the FC patterns of CP/CPPS patients. The explorations of the functional changes within longitudinal observation and after treatments could provide more information about the role of BG on the pathogenesis of CP/CPPS.

## Conclusion

In conclusion, this study directly supported the hypothesis that CP/CPPS was associated with abnormal function in the BG. Specifically, we found reduced rs-FC in the cortico-BG in CP/CPPS patients compared to healthy controls. The abnormal connectivity was associated with deficits in pain processing and micturition control in CP/CPPS. Based on the rs-FC of BG subregions, machine learning could be a reliable classifier in differentiating CP/CPPS patients and HCs. This current work provided neuroimaging evidence to improve our understanding of the neuropathological mechanisms of CP/CPPS.

## Data availability statement

The raw data supporting the conclusions of this article will be made available by the authors, without undue reservation.

## Ethics statement

The studies involving human participants were reviewed and approved by the Medical Ethics Review Board of Xi’an No.3 Hospital. The patients/participants provided their written informed consent to participate in this study.

## Author contributions

XL, JH, and MZ contributed to the conception and design of the study. W-JM, J-LL, JH, and W-XB contributed to the acquisition of the data. XN, H-NL, W-HD, and XL contributed to the analysis and interpretation of the data. X-YZ, XN, and XL contributed to the drafting of the article and critical revisions. All authors approved the submission of the manuscript for consideration.

## Footnotes

1
www.nitrc.org/projects/conn

2
https://www.nitrc.org/projects/mricron
