# Supplementary material for: The functional connectivity of the basal ganglia subregions changed in mid-aged and young males with chronic prostatitis/chronic pelvic pain syndrome
Source: Front Hum Neurosci. 2022 Sep 30;16:1013425. doi: 10.3389/fnhum.2022.1013425 (PMC9563619; doi:10.3389/fnhum.2022.1013425)
Supplement: Supplementary file 1 [file Data_Sheet_1.docx]

Supplementary Material

# Supplementary Figures and Tables

**Supplementary table 1** The clinical profiles of patients with CP/CPPS.

Note: the clinical profile of patients with CP/CPPS. NIH-CPSI, the National Institutes of Health chronic prostatitis symptom index. PCS, the Pain Catastrophizing Scale. NSAID, nonsteroidal anti-inflammatory drug.


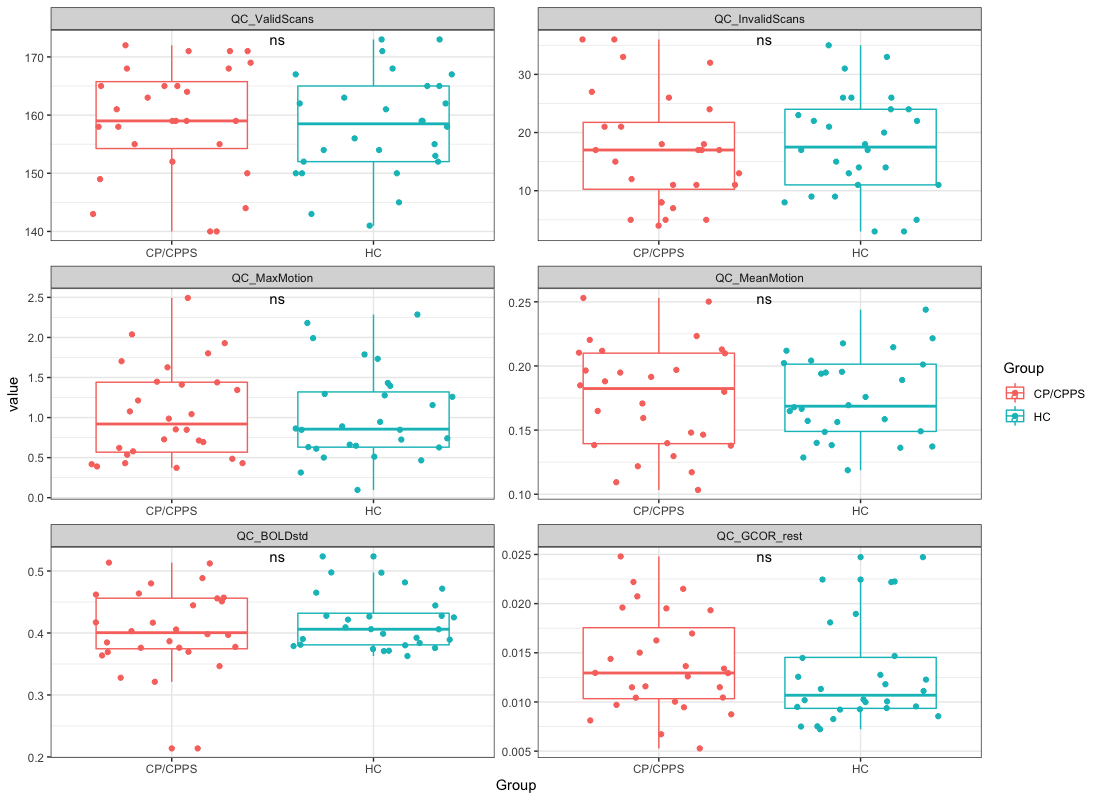


**Supplementary Figure 1** The variables related to head motion were no significant differences between two CP/CPPS and HC.

Note: ‘**QC_MaxMotion**’, ‘**QC_MeanMotion**’,‘**QC_ValidScans**’, and ‘**QC_InvalidScans**’ were automatically created after the outlier scan identification ART-based scrubbing step, which containing the maximum and mean inter-scan movement (framewise displacement), number of valid scans, and number of invalid scans, respectively. ‘**QC_BOLDstd**’ and ‘**QC_GCOR**’ were created in the denoising step and contained the global correlation index (average correlation coefficient between every pair of voxels across the entire brain) for each subject/condition. ns, no significant.


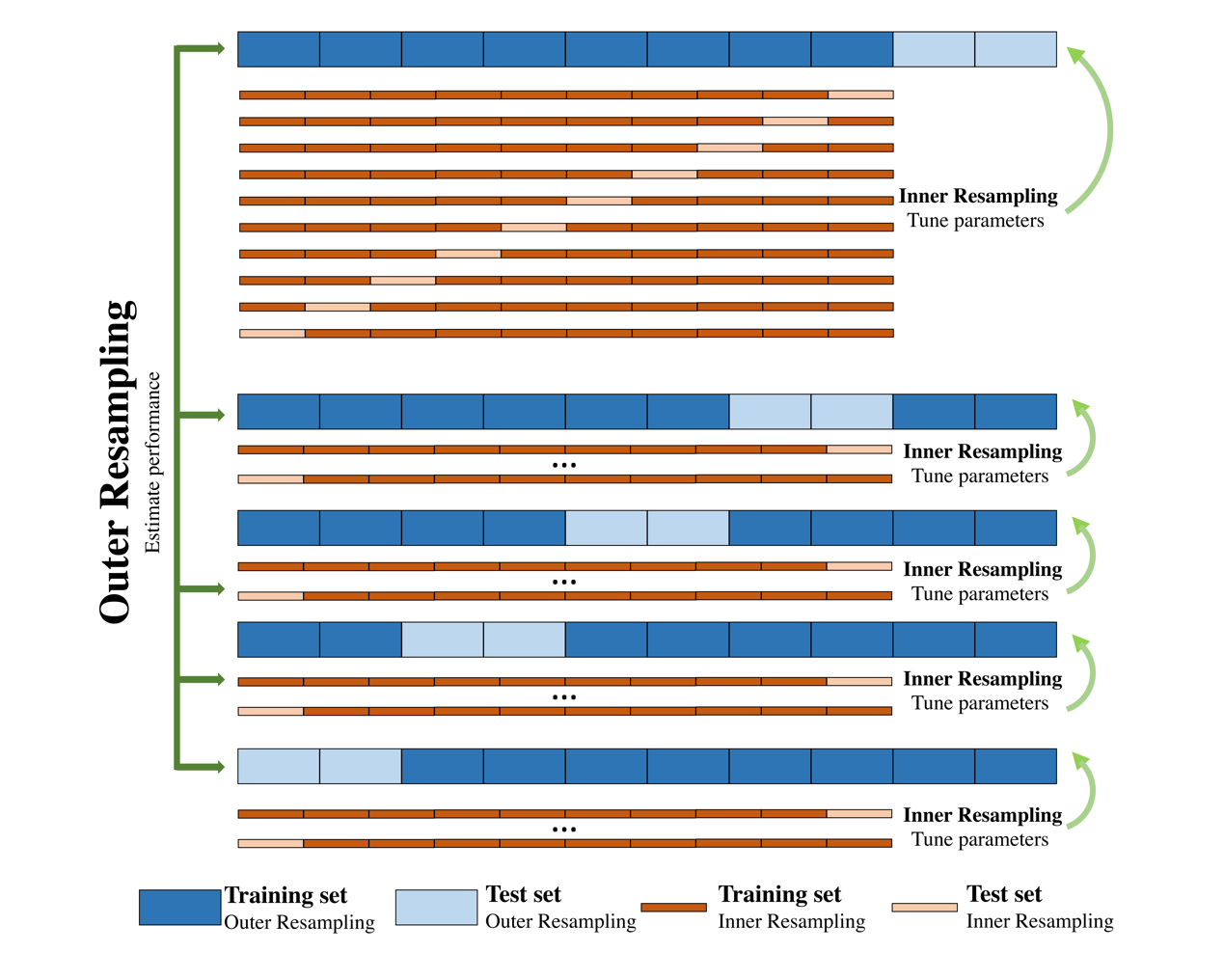


**Supplementary Figure 2.** Nested resampling. 10-fold inner resampling and 5-fold outer resampling.
